# Forecasting the Global Burden of Peripheral Artery Disease from 2021 to 2050: A Population-Based Study

**DOI:** 10.34133/research.0702

**Published:** 2025-07-01

**Authors:** Liling Deng, Chenzhen Du, Lu Liu, Yanzhong Wang, Haotian Gu, David G. Armstrong, Joseph L. Mills, Dirk Hochlenert, Huacong Deng, Junlin Ran, Yan Chen, Xiaoyan Jiang, Yu Ma, Qiu Chen, Wuquan Deng, on behalf of the “Flow and Toe” Research Team (FORT)

**Affiliations:** ^1^ School of Medicine, Diabetic Foot Medical Research Center, Department of Endocrinology, Chongqing University Central Hospital, Chongqing University, Chongqing 400014, China.; ^2^ School of Life Course and Population Health Sciences, King’s College London, London WC2R 2LS, UK.; ^3^ British Heart Foundation Centre, King’s College London, London WC2R 2LS, UK.; ^4^ Department of Surgery, Keck School of Medicine of University of Southern California, Los Angeles, CA 90033, USA.; ^5^ Division of Vascular Surgery and Endovascular Therapy, Department of Surgery, Baylor College of Medicine, Houston, TX 77030, USA.; ^6^ Ambulantes Zentrum für Diabetologie, Endoskopie und Wundheilung, Köln 50733, Germany.; ^7^ Diabetic Foot Diagnosis and Treatment Center, Department of Endocrinology, Hospital of Chengdu University of Traditional Chinese Medicine, Chengdu 610500, China.

## Abstract

Vascular disease is the leading cause of death worldwide. Predicting the burden of vascular disease and identifying modifiable key risk factors are critical for developing effective prevention strategies. This study aimed to project the global and regional burden of peripheral artery disease (PAD) from 2021 to 2050, with a specific focus on the impact of modifiable key risk factors and the potential benefits of their management. Compared to the 2021 Global Burden of Disease Study (GBD 2021), the number of PAD cases worldwide is projected to increase by 220% by 2050, reaching a staggering 360 million (95% uncertainty interval, 270 to 450). Age-standardized mortality is expected to double, while disability-adjusted life years (DALYs) are forecasted to rise from 19.7 to 33.1 per 100,000. Among individuals aged ≥65 years, PAD prevalence is projected to surge to 21.7% in women and 14.8% in men. Moreover, over 50% of PAD cases are expected to occur in low- and middle-income countries (LMICs). Metabolic diseases are anticipated to be the primary drivers of the rising PAD burden, with diabetes playing a key role in increasing PAD prevalence and severity. By effectively managing metabolic risk factors, age-standardized prevalence could be reduced by 36%, mortality by 17%, and DALYs by 10%. As metabolic risks, particularly diabetes, continue to rise alongside population aging, the global PAD burden is expected to increase substantially, especially in LMICs. Importantly, proactive metabolic risk management strategies have the potential to markedly alleviate the burden of vascular disease and reduce the growing geographic health disparities.

## Introduction

With global aging, industrialization, and urbanization, chronic noncommunicable diseases have risen dramatically, establishing peripheral artery disease (PAD) as a growing public health concern [1–3]. According to the Global Burden of Disease (GBD) 2021 estimates, over 113 million individuals worldwide are affected by PAD, a condition whose global prevalence may exceed that of coronary artery disease (CAD) [4]. PAD is associated with substantial morbidity, including varying levels of limb amputation, particularly among older adults [5], and is frequently accompanied by cardiovascular and cerebrovascular diseases, leading to a substantially increased risk of adverse vascular events and all-cause mortality, with a 5-year mortality rate estimate of at least 30% [1,6–8]. Furthermore, PAD imposes a substantial economic burden, with annual hospitalization costs in the United States alone reaching $6.31 billion [9].

Approximately 70% of PAD cases are attributed to modifiable risk factors such as smoking, obesity, diabetes, hypertension, and hyperlipidemia [2]. Recent GBD analyses highlight the escalating severity of metabolic risk factors, with diabetes alone projected to affect 1.31 billion people globally by 2050 [10,11]. Consequently, the global PAD burden is anticipated to increase substantially in the coming decades. While anti-smoking initiatives have demonstrated early success in reducing PAD risk, the potential impact of targeted interventions addressing major metabolic risk factors remains unquantified, and to date, no existing models integrate dynamic interactions between demographic shifts, socioeconomic determinants, and metabolic risk to project future PAD burden. Predicting the burden of PAD and identifying modifiable key factors are critical for developing effective prevention strategies.

Disease burden forecasting is crucial for guiding the strategic allocation of healthcare resources [12], anticipating the demands of an aging population, and identifying modifiable risk factors to mitigate adverse outcomes such as cardiovascular events, limb amputations, and mortality among PAD patients [7]. This study utilizes the GBD 2021 database to develop a dynamic prediction model, projecting the global burden of PAD [including prevalence, mortality, and disability-adjusted life years (DALYs)] across 204 countries and territories by 2050. Our approach incorporates key metabolic risk factors, demographic transitions, and socioeconomic indices to provide a comprehensive analysis of PAD burden under scenarios of medical intervention and non-intervention. These projections aim to supplement existing evidence by offering actionable insights into PAD prevention and management strategies in a rapidly changing global landscape.

## Results

In comparison, we estimated a substantial percentage increase of 220% in the total number of PAD cases globally between 2021 and 2050 (Table). In 2021, there were an estimated 113 [95% uncertainty interval (UI), 98 to 131] million individuals living with PAD worldwide, which is projected to increase to 189 (144 to 231) million by 2030, 273 (203 to 339) million by 2040, and 363 (270 to 454) million by 2050. We estimated that the age-standardized prevalence rate, after adjusting for population differences, would increase by 164%, from 1,441.0 (1,248.2 to 1,662.8) per 100,000 in 2021 to 3,803.6 (2,825.0 to 4,749.5) per 100,000 in 2050.

**Table.** Number/rate of PAD prevalence, deaths, and DALYs in 2021 and 2050 and percentage change (95% UI)

	Number of prevalence			Rate (per 100,000) of prevalence			Rate (per 100,000) of deaths			Rate (per 100,000) of DALYs		
	2021	2050 forecast	% Change	2021	2050 forecast	% Change	2021	2050 forecast	% Change	2021	2050 forecast	% Change
Global	113,711,840.6 (98,498,937.1–131,217,769.1)	363,296,638 (269,831,086.3–453,648,235.3)	220%	1,441.0 (1,248.2–1,662.8)	3,803.6 (2,825.0–4,749.5)	164%	0.9 (0.8–0.9)	1.8 (1.6–2.0)	100%	19.7 (16.1–25.9)	33.1 (28.6–37.8)	68%
Males	37,537,259.5 (32,491,217.7–43,499,076.2)	127,957,371.6 (78,600,224.9–172,901,667.6)	241%	948.1 (820.6–1,098.6)	2,679.1 (1,645.7–3,620.1)	183%	0.8 (0.8–1.0)	2.0 (1.8–2.2)	150%	19.0 (16.4–23.3)	34.3 (28.3–40.9)	81%
Females	76,174,581.1 (66,056,645.3–87,743,814.7)	235,339,266.3 (191,230,861.4–280,746,567.8)	209%	1,937.3 (1,680.0–2,231.6)	4,928.2 (4,004.5–5,879.1)	154%	0.9 (0.7–1.0)	1.7 (1.5–1.8)	89%	20.5 (15.5–29.0)	31.8 (28.9–34.7)	55%
Central Europe, eastern Europe, and central Asia	7,634,042.9 (6,588,506.3–8,866,102)	10,750,677.5 (8,065,809.1–13,737,491.5)	41%	1,827.06 (1,576.83–2,121.93)	5,180.8 (3,887–6,620.2)	184%	4.04 (3.68–4.38)	8.06 (5.75–10.93)	100%	76.09 (68.3–85.63)	122.6 (93.48–156.31)	61%
Central Asia	747,389.1 (643,468.4–872,739.3)	2,608,203 (2,069,696.5–3,158,525.8)	249%	780.08 (671.62–910.92)	4,249.7 (3,372.3–5,146.3)	445%	0.31 (0.27–0.36)	0.79 (0.51–1.18)	153%	9.24 (7.04–12.5)	19.04 (12.24–27.7)	106%
Central Europe	2,476,325.5 (2,131,442.5–2,863,685.2)	4,198,637.8 (2,912,554.3–5,518,214.7)	70%	2,148.4 (1,849.19–2,484.46)	8,649.5 (6,000.1–11,367.9)	303%	5.67 (5.06–6.2)	13.11 (9.86–17.11)	131%	97.74 (86.94–111.23)	179.62 (143.79–222.09)	84%
Eastern Europe	4,410,328.3 (3,832,268.2–5,113,474.1)	6,362,176.8 (5,600,895–7,153,365.4)	44%	2,133.07 (1,853.49–2,473.15)	6,519.1 (5,739–7,329.8)	206%	4.85 (4.38–5.37)	10.28 (7.13–14.66)	112%	95 (85.04–106.94)	162.11 (123.41–212.61)	71%
High income	41,984,904.8 (37,069,329.7–47,834,581.6)	74,873,428.4 (53,292,020.8–98,481,223.9)	78%	3,845.95 (3,395.67–4,381.8)	13,169.2 (9,373.3–17,321.4)	242%	3.1 (2.62–3.37)	6.89 (5.33–8.27)	122%	57.55 (47.95–71.55)	99.54 (79.62–122.2)	73%
Australasia	644,855.2 (555,212.9–750,912.8)	1,815,733.7 (1,272,348.6–2,372,490.5)	182%	2,082.77 (1,793.24–2,425.31)	9,474.7 (6,639.3–12,380)	355%	3.37 (2.81–3.76)	8 (5.92–10.23)	138%	48.1 (40.6–56.53)	92.29 (70.74–114.97)	92%
High-income Asia Pacific	6,051,973.8 (5,262,170.8–7,004,085.2)	11,697,090.4 (6,796,249.8–16,599,307.7)	93%	3,263.46 (2,837.57–3,776.88)	14,263.5 (8,287.4–20,241.3)	337%	1.36 (1.06–1.56)	3.34 (2.36–4.2)	145%	29.25 (21.45–40.76)	51.82 (37.17–70.28)	77%
High-income North America	16,414,011.6 (14,684,927.9–18,336,190.1)	31,082,622.6 (26,419,781.5–35,827,751)	89%	4,434.13 (3,967.03–4,953.39)	15,153 (12,879.8–17,466.3)	242%	3.32 (2.83–3.61)	7.42 (5.69–9.06)	124%	66.68 (56.85–81.48)	113.06 (92.04–140.52)	70%
Southern Latin America	1,316,214 (1,149,305.6–1,519,227.5)	3,621,391.6 (2,915,051.6–4,338,546.4)	175%	1,944.35 (1,697.78–2,244.24)	9,165.9 (7,378.2–10,981.1)	371%	0.68 (0.59–0.74)	1.92 (1.53–2.34)	183%	18.25 (13.77–26.17)	37.17 (27.54–50.48)	104%
Western Europe	17,557,850.2 (15,291,189.3–20,290,334.8)	33,646,886 (24,283,208–42,910,164.4)	92%	4,014.24 (3,496.01–4,638.97)	15,105.5 (10,901.8–19,264.2)	276%	4.01 (3.36–4.38)	8.4 (6.61–9.98)	110%	68.58 (56.92–84.85)	115.45 (93.41–140.63)	68%
Latin America and Caribbean	5,540,523 (4,779,073.6–6,437,943.7)	21,803,139.5 (18,237,681.5–25,629,518.2)	294%	932.56 (804.4–1,083.61)	5,996.8 (5,016.2–7,049.3)	543%	0.9 (0.8–0.97)	2.42 (1.6–3.49)	169%	19.54 (16.85–23.99)	42.13 (29.43–58.24)	116%
Andean Latin America	420,836.2 (362,414.9–491,143.5)	1,769,708.5 (1,543,026.3–2,028,490.8)	321%	636.35 (548.01–742.66)	3,961.1 (3,453.7–4,540.3)	522%	0.12 (0.1–0.15)	0.39 (0.27–0.56)	223%	4.9 (3.34–7.85)	12.08 (7.44–19.47)	146%
Caribbean	503,767.4 (435,558.9–583,347.1)	1,392,802.1 (1,261,005.1–1,534,590.7)	176%	1,061.49 (917.76–1,229.17)	5,507.8 (4,986.6–6,068.5)	419%	2.27 (1.97–2.52)	5.32 (3.81–7.25)	135%	40.22 (35.18–46.61)	78.8 (58.24–103.13)	96%
Central Latin America	2,294,591.8 (1,976,827.8–2,663,663.9)	10,258,913.8 (8,226,820.4–12,496,077.7)	347%	906.95 (781.35–1,052.82)	6,092.4 (4,885.6–7,421)	572%	0.39 (0.34–0.43)	1.29 (0.83–1.87)	231%	10.46 (7.99–14.83)	26.86 (17.18–38.77)	157%
Tropical Latin America	2,321,327.6 (2,003,435.6–2,698,875.8)	9,513,379.4 (7,439,807.5–11,733,279)	310%	1,020.25 (880.53–1,186.19)	7,597 (5,941.2–9,369.8)	645%	1.41 (1.23–1.53)	3.74 (2.44–5.42)	166%	29.58 (26.1–34.42)	61.36 (42.62–84.86)	107%
North Africa and Middle East	4,446,618.3 (3,813,054.9–5,179,552.8)	30,112,245 (20,956,657.6–39,569,729.6)	577%	713.74 (612.05–831.39)	6,816.2 (4,743.8–8,957.1)	855%	0.16 (0.12–0.18)	0.49 (0.3–0.7)	212%	5.91 (4.26–8.81)	14.62 (8.51–22.9)	147%
South Asia	12,208,764.3 (10,456,449.3–14,263,158.2)	41,586,662 (36,620,556.4–47,126,270.8)	241%	661.16 (566.26–772.41)	4,008 (3,529.4–4,541.9)	506%	0.16 (0.1–0.26)	0.61 (0.33–1.11)	282%	6.03 (3.84–9.46)	16.08 (9.15–26.29)	167%
Southeast Asia, east Asia, and Oceania	38,554,836.6 (33,102,251.2–44,952,718.1)	111,261,678.7 (95,710,764.3–127,380,574.5)	189%	1,764.52 (1,514.97–2,057.33)	10,352.2 (8,905.3–11,852)	487%	0.14 (0.11–0.18)	0.53 (0.34–0.75)	276%	10.85 (6.37–18.99)	27.48 (14.83–48.65)	153%
East Asia	29,614,169.9 (25,445,754.5–34,511,057.6)	178,252,404.4 (69,904,626.4–282,608,107.2)	502%	2,010.77 (1,727.74–2,343.27)	26,757.4 (10,493.4–42,422.3)	1,231%	0.16 (0.13–0.2)	0.66 (0.41–0.94)	310%	12.08 (6.96–21.27)	32.5 (17.38–57)	169%
Oceania	83,822.6 (71,436.3–98,166.6)	381,889.6 (337,189.8–433,693.2)	356%	601.85 (512.91–704.84)	3,409.9 (3,010.8–3,872.5)	467%	0.06 (0.03–0.08)	0.11 (0.06–0.2)	100%	4.56 (2.63–7.59)	8.42 (4.48–14.2)	85%
Southeast Asia	8,856,844.1 (7,562,694.1–10,344,756.3)	41,782,520.4 (27,907,420.1–55,542,613.8)	372%	1,268.34 (1,083.01–1,481.41)	10,514.5 (7,022.8–13,977.2)	729%	0.1 (0.08–0.13)	0.34 (0.22–0.49)	233%	8.39 (4.96–14.25)	20.02 (11.34–34.83)	139%
Sub-Saharan Africa	3,342,150.7 (2,848,718.7–3,903,326.7)	27,100,452.4 (15,303,460.8–39,388,567.9)	711%	294.94 (251.4–344.46)	2,505.5 (1,414.9–3,641.6)	750%	0.42 (0.29–0.71)	1.04 (0.64–1.84)	152%	9.75 (6.76–15.59)	21.06 (13.4–34.79)	116%
Central sub-Saharan Africa	366,670.6 (312,142.6–431,035.3)	2,401,622.3 (2,016,936.5–2,814,791.5)	555%	267.78 (227.96–314.79)	1,932.5 (1,623–2,265)	622%	0.45 (0.25–0.76)	1.28 (0.66–2.22)	182%	11.02 (6.43–18.24)	26.93 (14.93–44.89)	144%
Eastern sub-Saharan Africa	1,087,519.8 (925,851.4–1,276,944.8)	10,814,740.7 (6,393,746.7–15,257,667)	894%	255.23 (217.29–299.68)	2,663.7 (1,574.8–3,757.9)	944%	0.31 (0.18–0.58)	0.87 (0.46–1.67)	179%	7.37 (4.48–13.16)	18.02 (10.33–32.19)	145%
Southern sub-Saharan Africa	553,683.3 (473,669–647,935.5)	2,431,716.9 (1,978,138.7–2,918,542.7)	339%	689.49 (589.85–806.86)	4,219.9 (3,432.8–5,064.7)	512%	1.19 (1.05–1.34)	2.86 (2.34–3.44)	140%	30.47 (26.4–35.1)	62 (50.47–74.43)	103%
Western sub-Saharan Africa	1,334,276.9 (1,141,625.4–1,558,018.9)	7,761,664.9 (7,257,369.7–8,388,792.1)	482%	272.4 (233.07–318.07)	1,572.1 (1,470–1,699.1)	477%	0.37 (0.22–0.75)	0.94 (0.5–1.83)	157%	8.08 (4.97–14.75)	17.89 (10.34–33.15)	121%

**Fig. 1.**
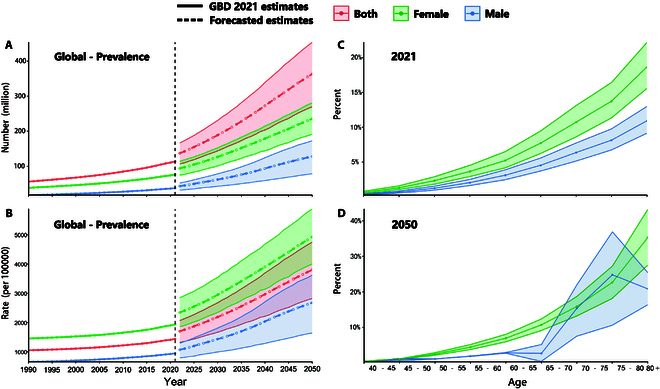
Estimated trends in the global all-age number of cases (A) and age-standardized PAD prevalence (B), with 95% UIs, 2022–2050; global prevalence of PAD by age group and sex in 2021 (C) and 2050 (D), with 95% UIs.

In 2021, there were more females with PAD than males with PAD globally, with a female-to-male ratio of 2.03. We anticipate that this pattern will decrease slightly by 2050 due to a higher growth rate of PAD cases among males, resulting in a female-to-male ratio of 1.84 (Table and Fig. 1). By 2050, the projected prevalence rates of PAD among females aged 40 to 59, 60 to 69, 70 to 79, and ≥80 years would be 2.1% (95% UI, 1.8 to 2.4), 8.7% (7.4 to 10.1), 19.0% (15.5 to 22.6), and 35.3% (27.5 to 43.2), respectively. The corresponding prevalence rates for males in these age groups are projected to be 1.0% (0.9 to 1.1), 2.8% (1.7 to 4.0), 19.6% (8.9 to 28.6), and 20.8% (16.4 to 25.4) (Table and Fig. 1).

**Fig. 2.**
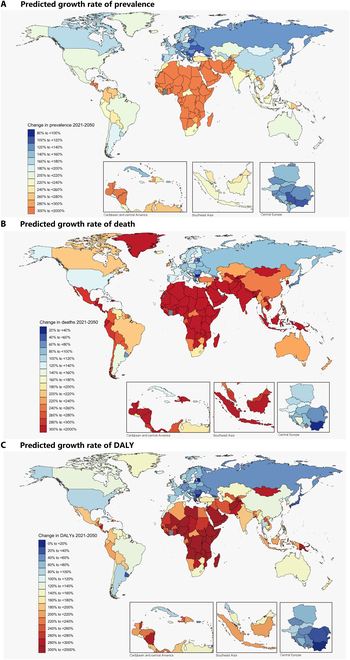
Percentage change between 2021 and 2050 in all-age number of prevalence (A), deaths (B), and DALYs (C) with PAD by country.

Between 2021 and 2050, we estimated that the highest percentage change in total PAD cases would occur in sub-Saharan Africa (711% [95% UI, 437 to 909]), while the lowest percentage increases were projected in Central Europe, Eastern Europe, and Central Asia (41% [22 to 55]). Regarding age-standardized prevalence, the highest percentage change was projected in North Africa and the Middle East (855% [675 to 977]), whereas Central Europe, Eastern Europe, and Central Asia would exhibit the lowest rate of change (184% [147 to 212]) (Table). When examining the projected percentage changes in age-standardized PAD prevalence by country or region, substantial heterogeneity was evident. While increases were anticipated across all countries and regions, high-income regions such as Eastern Europe (206% [95% UI, 176 to 295]) were projected to experience the lowest increases, whereas the highest increases were expected in East Asia (1,231% [507 to 1,710]), eastern sub-Saharan Africa (944% [625 to 1,154]), and North Africa and the Middle East (855% [675 to 977]) (Table and Fig. 2).

**Fig. 3.**
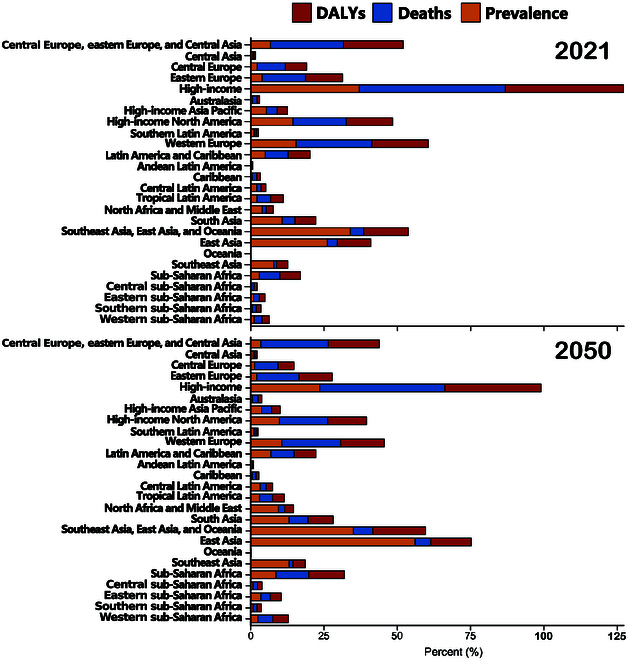
Proportion of prevalence, deaths, and DALYs in different countries or regions.

The age-standardized mortality rate for PAD was expected to increase by 100% (95% UI, 116 to 117) from 0.9 (0.8 to 0.9) per 100,000 in 2021 to 1.8 (1.6 to 2.0) per 100,000 in 2050 (Table and Fig. 2). Age-standardized DALYs were projected to increase by 67% (46 to 78), rising from 19.7 (16.1 to 25.9) per 100,000 in 2021 to 33.1 (28.6 to 37.8) per 100,000 in 2050, with a higher increase among males (Table and Fig. 2). Further analysis of the projected percentage changes in age-standardized PAD mortality by country or region indicated that South Asia (282% [95% UI, 215 to 331]) would experience the highest increase in mortality by 2050. Although the lowest increases were projected in Oceania (100% [71 to 146]) and western Europe (110% [97 to 128]), mortality rates in these regions would be twice those reported in 2021. Projections for age-standardized DALYs indicated that the highest increases were in South Asia (167% [138 to 178]) and Central Latin America (157% [115 to 161]), while the lowest increases were expected in western Europe (68% [64 to 66]) and high-income North America (70% [62 to 72]) (Table and Figs. 2 and 3). At the national level, the highest projected increases by 2050 are in Qatar (1,691% [1,467 to 1,875]) and the United Arab Emirates (1,553% [1,263 to 1,884]), while the lowest projected increases are in Bulgaria (9% [−4 to 16]) and Estonia (16% [−2 to 31]).

**Fig. 4.**
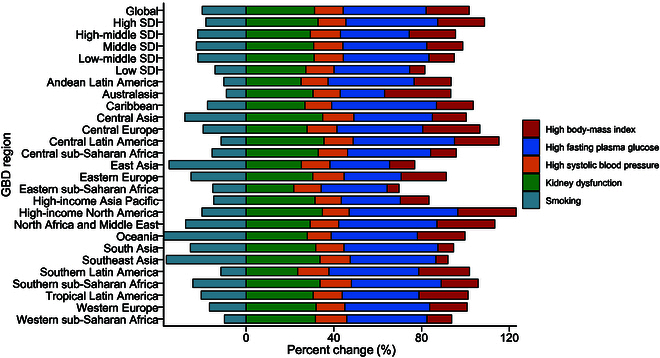
Decomposition of percentage change in the number of individuals with PAD between 2021 and 2050 globally and by world region.

Decomposition of the projected percentage changes in PAD cases suggested that metabolic risks would substantially contribute to the overall increase in prevalence, nearly matching the impact of population aging. In most regions, the increase in PAD cases was attributed to GBD risk factors including high fasting plasma glucose, high body mass index, kidney dysfunction, and high systolic blood pressure. In contrast, smoking-related PAD cases were projected to decline by 2050, primarily due to global anti-smoking initiatives. Although metabolic risks contributed similarly to the projected increase in PAD prevalence at the global level, different patterns are projected to be observed at the regional level. High-income North America was expected to experience the largest contribution of metabolic risks to PAD prevalence, while eastern sub-Saharan Africa was expected to see the smallest contribution (Fig. 4).

**Fig. 5.**
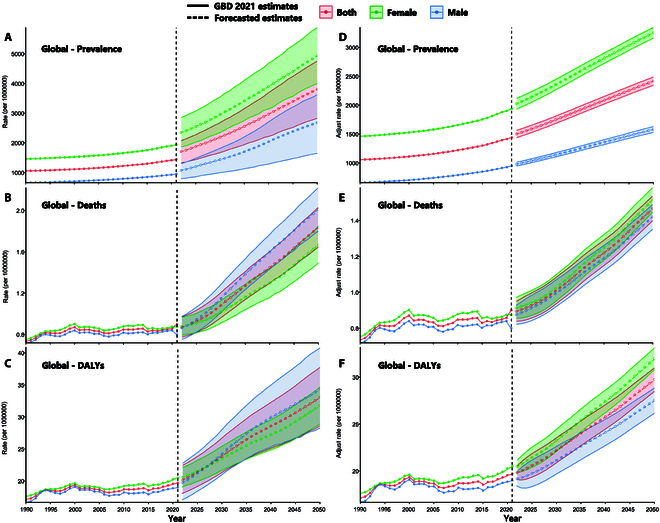
Estimated trends in the global age-standardized PAD prevalence (A), deaths (B), and DALYs (C) under reference conditions; estimated trends in the global age-standardized PAD prevalence (D), deaths (E), and DALYs (F) under improved metabolic risk conditions, with 95% UIs, 2022–2050.

Based on these findings, further projections indicated that substantial reductions in PAD burden can be achieved by improving major metabolic risk factors. We constructed a counterfactual baseline scenario that assumes strict implementation of risk management measures globally. Specifically, the age-standardized prevalence of PAD could decrease by 36%, from 3,803.6 (95% UI, 2,825.0 to 4,749.5) per 100,000 to 2,416.4 (2,344.9 to 2,487.9) per 100,000. Mortality could be reduced by 17%, from 1.8 (1.6 to 2.0) per 100,000 to 1.5 (1.4 to 1.5) per 100,000, and DALYs could be reduced by 10%, from 33.1 (28.6 to 37.8) per 100,000 to 29.8 (28.5 to 31.1) per 100,000 (Fig. 5).

## Discussion

Our study provides a comprehensive projection of the global PAD burden, including prevalence, mortality, and DALYs, across 204 countries and regions up to 2050. These projections address gaps in previous studies by providing a detailed assessment of the potential PAD burden resulting from increasing global metabolic risks. While the effectiveness of global anti-smoking campaigns has contributed to a reduction in age-standardized PAD rates, these gains are counterbalanced and may be surmounted by population aging and the increasing prevalence of metabolic diseases. If current trends continue without effective interventions, the global PAD burden is projected to double by 2050.

We estimate that the total number of PAD cases will increase by 220%, potentially affecting 360 million (95% UI, 270 to 450 million) individuals by 2050. While the overall number of cases in females is expected to exceed that in males, the growth rate in males is projected to outpace that in females [2,13]. This finding is consistent with previous reports and suggests that the higher growth rate in males is likely due to higher smoking rates, while the higher total case number in females reflects their longer life expectancy. Biological factors may also contribute to gender differences in PAD prevalence. First, the differences in sex hormones may affect arterial compliance. Second, increased immune molecules, immune cells, and platelets in women with PAD may contribute to the higher female prevalence and altered pathogenesis of the disease. In addition, women have higher resistance to antiplatelet agents [14–16]. The elderly population remains the most vulnerable group, with PAD prevalence among those aged 60 years and above projected to rise from 8.0% in 2021 to 15.2% in 2050. Additionally, as ^2^/_3_ of the global population 60 years of age and above will reside in LMICs by 2050 [17], it is expected that over half of PAD patients will live in these regions. The increase in DALYs highlights the substantial healthcare resource utilization and disability risk faced by PAD patients in these regions.

While population aging is a key driver of PAD incidence, modifiable metabolic risk factors and unhealthy behaviors contribute to 70% of PAD cases globally [2]. Public health interventions targeting these risk factors have already been prioritized by health organizations, yet the implementation and effectiveness of these measures remains suboptimal [18]. Among the metabolic risks, diabetes stands out as the most significant contributor to PAD, accounting for 37.7% of age-standardized DALYs.

The link between diabetes and PAD is well established, with diabetic patients being 2 to 3 times more likely to develop PAD, and nearly half of diabetic foot ulcer patients suffering from the condition [19–22]. Other major risk factors, such as obesity, hypertension, and hyperlipidemia, also substantially contribute to PAD burden. Despite the high prevalence of these conditions, disparities in diagnosis, treatment, and control remain widespread due to unequal healthcare resource distribution [23–25]. Furthermore, studies have shown that important interactions and mediating effects exist among the main risk factors. For example, diabetes and obesity may synergistically exacerbate the risk of developing PAD by promoting systemic inflammatory responses and endocrine imbalances. In addition, smoking, as an independent behavioral risk factor, may exert long-term effects by promoting atherosclerosis and, together with metabolic abnormalities, synergistically deteriorate vascular function [2]. By applying the population attributable fraction (PAF) model, this study has, to some extent, reflected the cumulative effects of these interacting risk factors. However, we acknowledge that further mediation effect analyses are needed to more precisely quantify the pathways and proportions of the influence among these factors, thereby providing theoretical support for targeted prevention strategies.

Chronic kidney disease (CKD), a nontraditional metabolic risk factor, also plays a crucial role in PAD development, with a 29.0% contribution to PAD-related age-standardized DALYs. CKD patients with PAD are at increased risk for limb amputations, cardiovascular events, mortality, and all-cause mortality [26]. Our study highlights that those unhealthy behaviors, such as smoking, physical inactivity, and high sodium intake, account for over 30% of PAD-related age-standardized mortality. Smoking remains the primary behavioral risk factor. Despite progress in smoking cessation campaigns, the long-term impact of smoking on PAD risk persists, with former smokers taking decades to reach the same risk level as nonsmokers [27]. For example, a recent community-based cohort study indicates that former smokers may take up to 30 years to reach the same PAD risk level as nonsmokers [28]. Additionally, the burden associated with physical inactivity and high sodium intake is expected to rise.

Our projections suggest that the combined effects of aging and increasing metabolic risks will partially offset the benefits of smoking cessation and medical interventions targeting metabolic conditions. Under a scenario where medical interventions effectively control metabolic risk factors, we estimated a 36% reduction in age-standardized PAD prevalence, a 17% reduction in mortality, and a 10% reduction in DALYs by 2050. These finding highlights that more than 30% of PAD cases could be preventable through effective risk factor management.

The limited awareness of PAD among healthcare professionals and the public, particularly in socioeconomically disadvantaged regions, contributes to delayed diagnosis and underdiagnosis [29]. Only 10% to 30% of PAD patients present with classic symptoms of intermittent claudication, which complicates early detection and timely treatment [30]. The resting ankle brachial index (ABI), a simple, noninvasive physiological test, remains as the primary method for initial diagnosis of PAD. In addition, other diagnostic testing for PAD is performed to supplement the ABI and includes exercise ABI testing, segmental pressures, leg pressures and Doppler waveforms, toe branchial index, and perfusion imaging [14]. Despite the evidence from high-quality clinical trials, PAD remains under-recognized compared to CAD, leading to suboptimal adherence to guideline-recommended therapies. Global treatment rates for PAD remain low, especially in low-sociodemographic index (SDI) regions, with substantial gaps in the prescription of antiplatelet therapy, statins, and proprotein convertase subtilisin/kexin type 9 inhibitors [31,32]. Furthermore, supervised exercise therapy, a first-line treatment, has demonstrated efficacy comparable to that of endovascular interventions. However, its implementation is hampered by inadequate healthcare infrastructure, particularly in resource-limited settings [33].

Our estimates can inform the planning of resources and healthcare services needed to address the growing PAD burden. Currently, substantial gaps exist globally in PAD awareness, diagnosis, and treatment. Diagnostic and treatment rates are lower in low- and middle-income regions than in high-income countries, where PAD management resources are more readily available. Efforts to address these disparities must include increasing public and healthcare provider awareness, improving access to diagnostic services, and ensuring equitable distribution of PAD management resources [31]. Government health agencies must take a leading role in strengthening PAD-related policies and expanding treatment options, especially in regions with limited healthcare infrastructure. By supporting comprehensive public health strategies and emphasizing the importance of managing metabolic risk factors, we can reduce the future burden of PAD and improve health outcomes globally.

This study has several limitations. Our analysis relies on data from the GBD 2021 study, which integrates estimates of PAD prevalence and mortality from diverse sources. Variations in methodology and data availability across regions may lead to discrepancies between GBD estimates and national or subnational data [10]. However, our focus was on projecting future trends rather than comparing absolute values across datasets. In addition, because the forecasting model is based primarily on GBD historical data and established assumptions, there are inevitably uncertainties introduced by technological advances, healthcare policy adjustments, and socioeconomic shifts. With the further development of the prediction model in the future, this uncertainty may be reduced. For example, dynamic adjustment mechanism is introduced to capture the changes of external environmental factors in a more timely manner and improve the sensitivity and accuracy of the prediction model. The projection of PAD prevalence using PAFs for GBD risk factors is based on assumed causal relationships. If noncausal factors influence the estimates, the projections may over- or underestimate true prevalence. Additionally, the estimation of PAD prevalence within the GBD framework is constrained by limited and heterogeneous data, as population-based studies often lack standardized diagnostic methods. Finally, while this study offers valuable insights into the future PAD burden, it highlights the need for more robust epidemiological research. Standardized diagnostic methods and exploration of novel therapies, such as GLP-1 receptor agonists (GLP1RAs), are essential. Retrospective studies suggest that GLP1RA may reduce the risk of major limb events in diabetic patients [34]. Recent evidence from randomized trials on PAD indicated that semaglutide increased walking distance in patients with symptomatic PAD and type 2 diabetes [35]. Future cardiovascular research should prioritize PAD as a key endpoint to enhance burden estimates and guide prevention strategies.

## Conclusion

Despite its limitations, this study is the first to offer comprehensive projections of the global burden of PAD, providing detailed forecasts for prevalence, mortality, and DALYs. Unlike previous studies that focused primarily on regional estimates, we utilized national-level data from the 2021 GBD dataset to project PAD prevalence by 2050 across countries, world regions, and SDI. By integrating projections of key metabolic risk factors and modeling their impact on PAD prevalence from 1990 to 2021, our analysis provides a more nuanced understanding of the future burden of PAD. Additionally, we employed decomposition analysis to quantify the relative contributions of population growth, aging, and changes in risk factors to the projected trends.

The granularity of our national-level estimates offers policymakers valuable insights into the anticipated rise in PAD cases and the underlying drivers of these increases within specific geographic and socioeconomic contexts. This information is critical for public health planning, particularly in ensuring adequate resource allocation to meet the growing demands of PAD patients and their caregivers. As population growth and aging emerge as major contributors to the increasing burden, urgent research is needed to develop disease-modifying therapies, cost-effective prevention strategies, and interventions targeting modifiable risk factors.

Our study provides a global perspective on PAD projections up to 2050, spanning 204 countries and territories and examining the interplay between metabolic risk factors and disease burden. We highlight the dynamic shifts in PAD prevalence across geographic regions, income groups, age categories, and genders while also exploring the potential impact of effective interventions targeting metabolic risk factors. These findings underscore the potential to substantially curb the rise in PAD prevalence through proactive public health measures, despite the challenges posed by an aging population.

By presenting a robust framework for predicting the future PAD burden, this study provides actionable insights for policymakers, healthcare planners, and researchers. Expanding healthcare service capacity, improving access to cost-effective treatments, and maximizing adherence to evidence-based strategies for PAD management should be prioritized to address the growing global burden. Furthermore, the identification of novel risk factors and the development of innovative interventions remain critical to mitigating the impact of PAD in the decades to come.

## Methods

### Overview

Using GBD 2021 estimates as input data, we projected the PAD burden across 204 countries and regions from 2022 to 2050. Our forecasting framework builds on the methodology established by Foreman et al. [36] and incorporates refinements to predict independent health drivers and their associations with specific health outcomes. The prevalence prediction framework integrates key drivers such as sociodemographic characteristics by location, SDI values, gender-age-specific risk factor exposures (e.g., smoking and metabolic risks), and age-specific population proportions. Prevalence and mortality attributable and non-attributable to GBD risk factors were modeled, with additional residual drift from other causes incorporated into the estimates [10].

### Data sources

The GBD 2021 study systematically reviewed globally representative data sources for PAD prevalence, mortality, and DALYs. Data visualization tools and the GBD protocol are publicly accessible, with historical population data obtained from the World Health Organization. Future population forecasts were derived using the method proposed by Vollset et al. [17] All concepts, analytical structures, and detailed methodologies related to GBD risk factors have been previously described and documented in prior GBD publications [2,10].

### Risk factor forecasts

We evaluated the influence of 6 primary risk factors for PAD [2], as identified in previous study: smoking, high fasting plasma glucose, high body mass index, kidney dysfunction, high sodium intake, and lead exposure. Additionally, we considered other factors including low physical activity, alcohol consumption, dietary patterns, and air pollution. Multivariate regression was applied to address potential correlations among risk factors, ultimately grouping them into 2 broad categories: metabolic risks and behavioral risks. Summary exposure value (SEV) forecasts were estimated using historical trends and weighted averages.

### The prevalence forecasts: Attributable to independent risk factors

We forecasted the combined effect of GBD risk factors by calculating the proportion of PAD prevalence attributable to each risk factor. Using both historical and predicted SEV values (1990–2021) along with their associated relative risk estimates, we estimated the PAF for each risk factor based on GBD 2021 data. Subsequently, we estimated the joint PAF for 2 risk categories and computed location-, age group-, and gender-specific scalars for each prediction year.

$$\text{Scalar}=\frac{1}{1-PAF}$$ (1)

Detailed methodologies for PAF estimation, risk factor mediation, and scalar computation are available in prior GBD projection studies [10,17].

### Prevalence forecasts: Not attributable to independent risk factors

The GBD risk-deleted prevalence was estimated by dividing total PAD prevalence by the risk factor scalar. To refine our prediction model, we evaluated associations between risk factors and PAD prevalence (logit-transformed), employing gender-stratified models adjusted for 5-year age groups and global regions. Covariates associated with PAD prevalence estimates for both males and females, with effect directions consistent with those reported by the Lancet Commission Report, were incorporated into the model [37]. Additionally, the SDI was included as a covariate, with SDI projections derived by calculating the weighted average of historical changes.

Gender-stratified linear regression models were used to predict logit-transformed, risk-deleted PAD prevalence, incorporating 5-year age groups, global regions, and SDI as covariates. To account for uncertainty in input estimates, we performed 2,000 model iterations, drawing from the distributions of each input variable. Residual trends unexplained by the covariates were modeled using a random walk (autoregressive integrated moving average, ARIMA[0,1,0]), with predicted residuals added to the final prevalence estimates [10]. We utilized a grid search over possible combinations of autoregressive (*p*), differencing (*d*), and moving average (*q*) orders, guided by information criteria such as the Akaike information criterion (AIC) and the Bayesian information criterion (BIC). This process ensured that the chosen model minimizes the information loss while maintaining parsimony.

$$\begin{array}{c} \left(\text{Logit}{\left(\text{prevalance}\right)}_{sex}\right)=\beta_{0}+\beta_{1}SDI+\sum_{i=2}^{12}\beta_{i}Age_{i}\\ \;+\sum_{i=13}^{204}\beta_{i}{\text{Country}}_{i}+\alpha \end{array}$$ (2)

### Comprehensive prevalence forecasts

The total PAD prevalence was projected as the product of risk-deleted prevalence and forecasted risk factor scalar. To ensure consistency between historical and future means and UIs, we applied an intercept shift to the predicted logit-transformed prevalence. Total PAD cases were calculated by multiplying prevalence projections by population forecasts. Using Das Gupta decomposition methods, we quantified the contributions of population growth, aging, and nondemographic factors to changes in PAD cases from 2021 to 2050 [37,38].

### Death and DALY forecasts

The forecasting framework for PAD-related mortality and DALYs mirrored that used for prevalence. This included (a) baseline mortality modeled using population characteristics, SDI, regions, and gender; (b) gender- and age-specific projections of risk factor exposure by location; and (c) a random walk model with attenuated drift to account for residual mortality trends.

### Improved behavioral and metabolic risks

This analysis refers to the hypothetical scenario of the GBD Foresight Visualization tool. Exposure to all dietary risk factors as well as high low-density lipoprotein cholesterol, body mass index, fasting plasma glucose, and systolic blood pressure are eliminated by 2050. (https://vizhub.healthdata.org/gbd-foresight/).

### Analytical tools

All analyses were conducted using Python (version 3.12.7) and R (version 4.4.2).

## Data Availability

Prevalence, deaths, and DALYs of peripheral artery disease from GBD 2021 are available for download via the GBD Results Tool (https://vizhub.healthdata.org/gbd-results/).

## Appendix A

### Members of the “Flow and Toe” Research Team (FORT)

Dongfeng Tang, Canlong Wang, Lidan Liu, Zhangrong Xu, Qingzhong Xiao, Yun Gao, Johnson Boey, Cheng Yang, Anxin Li, Shunli Rui, Zixiao Duan, Yi Yuan, Charareh Pourzand, Lisha Sun, Ya Liu, Rijian Song, Wenxin Wang, Zhongming Wu, Xiaoyan Yi, Lin Li, Li Zhang, Mengchen Zou, Peiyuan Huang, Zhihui Dong, Jiangang Lu.

### Affiliations

School of Life Course and Population Health Sciences, King’s College London, London, UK (D.T., C.W., L. Liu); Diabetes Centre, The 306th Hospital of PLA, Beijing, China (Z.X.); Centre for Clinical Pharmacology and Precision Medicine, William Harvey Research Institute, Faculty of Medicine and Dentistry, Queen Mary University of London, London, UK (Q.X.); Department of Endocrinology and Metabolism, Diabetic Foot Care Center, West China Hospital, Sichuan University, Chengdu, Sichuan, China (Y.G.); Department of Podiatry, National University Hospital Singapore, Singapore, Singapore (J.B.); Diabetic Foot Medical Research Center, Department of Endocrinology, Chongqing University Central Hospital, Chongqing University, Chongqing, China (C.Y., A.L., S.R., Z. Duan, Y.Y.); Department of Life Sciences, University of Bath, Bath, UK (C.P.); Department of Endocrinology, Hospital of Chengdu University of Traditional Chinese Medicine, Chengdu, China (L.S., Y.L.); Charles Institute of Dermatology, School of Medicine, University College Dublin, Dublin 4, Ireland (R.S., W.W.); Department of Endocrinology, Shandong Provincial Hospital Affiliated to Shandong First Medical University, Shandong First Medical University, Jinan, Shandong, China (Z.W.); ULB Center for Diabetes Research, Medical Faculty, Université Libre de Bruxelles, Brussels, Belgium (X.Y.); Department of Endocrinology, Zhejiang University School of Medicine Sir Run Run Shaw Hospital, Hangzhou, China (L. Li); Guang ’anmen Hospital, China Academy of Chinese Medical Sciences, Beijing, China (L.Z.); Department of Endocrinology and Metabolism, Nanfang Hospital, Southern Medical University, Guangzhou, China (M.Z.); MRC Integrative Epidemiology Unit (IEU), Bristol Medical School, University of Bristol, Oakfield House, Oakfield Grove, Bristol, UK (P.H.); Departments of Vascular Surgery of Zhongshan Hospital, Fudan University, Shanghai, China (Z. Dong); Department of Endocrinology and Metabolism, Chongqing University Fuling Hospital, Chongqing University, Chongqing, China (J.L.).
